# An Update on Microbial Interventions in Autism Spectrum Disorder with Gastrointestinal Symptoms

**DOI:** 10.3390/ijms252313078

**Published:** 2024-12-05

**Authors:** Rachel J. Moreno, Paul Ashwood

**Affiliations:** 1 Department of Medical Microbiology and Immunology, University of California, Davis, CA 95616, USA; 2 The M.I.N.D. Institute, University of California, Davis, CA 95817, USA

**Keywords:** autism, prebiotics, synbiotics, probiotics, diet, fetal microbiota transplant, FMT, antibiotics, inflammation, behavior, gut, brain–gut, gastrointestinal symptoms, GI, autism spectrum disorder, ASD

## Abstract

In the United States, autism spectrum disorder (ASD) affects 1 in 33 children and is characterized by atypical social interactions, communication difficulties, and intense, restricted interests. Microbial dysbiosis in the gastrointestinal (GI) tract is frequently observed in individuals with ASD, potentially contributing to behavioral manifestations and correlating with worsening severity. Moreover, dysbiosis may contribute to the increased prevalence of GI comorbidities in the ASD population and exacerbate immune dysregulation, further worsening dysbiosis. Over the past 25 years, research on the impact of microbial manipulation on ASD outcomes has gained substantial interest. Various approaches to microbial manipulation have been preclinically and clinically tested, including antibiotic treatment, dietary modifications, prebiotics, probiotics, and fecal microbiota transplantation. Each method has shown varying degrees of success in reducing the severity of ASD behaviors and/or GI symptoms and varying long-term efficacy. In this review, we discuss these microbiome manipulation methods and their outcomes. We also discuss potential microbiome manipulation early in life, as this is a critical period for neurodevelopment.

## 1. Introduction

Autism spectrum disorder (ASD) is defined as having restricted interests and atypical social and communicative behaviors [1]. Individuals with ASD also face a suite of co-morbidities, with the most prominent being gastrointestinal issues (GI) which include abdominal pain, constipation, and diarrhea [2]. A suggested reason for the increased prevalence of GI issues is microbial dysbiosis—a deviation of commensal microbial abundance and function—within the GI tract [3]. The most pre-dominate phyla of commensal gut microbes in humans include *Bacillota* (previously known as *Firmicutes)* and *Bacteroidetes*, followed by smaller populations composed of *Actinobacteria*, *Proteobacteria*, and *Verrucomicrobia* [4,5]. In gut biopsies from ASD children, shifts in the abovementioned phyla occur, with increased *Bacillota*, namely those part of the class *Clostridia*, and decreased *Bacteroidetes* [6]. This is opposite to what is found in stool samples, as *Bacteroidetes*, *Proteobacteria* and *Actinobacteria* are higher in severe ASD cases while *Bacillota* is lower [7,8]. In mice that received fecal transplants from ASD children, ASD-like behavior, including reduced socialness and increased anxiety was observed [9]. However, in this instance, *Actinobacteria* was significantly reduced in mice that received ASD donor stool, as well as *Candidative* S., whereas the *Temericutes* phyla was increased [9]. Based on these and similar studies, it has been hypothesized that microbial dysbiosis is one of the contributing factors in ASD etiology or pathophysiology, and therefore manipulation of the microbiome may be of critical clinical importance.

**Figure 1:**
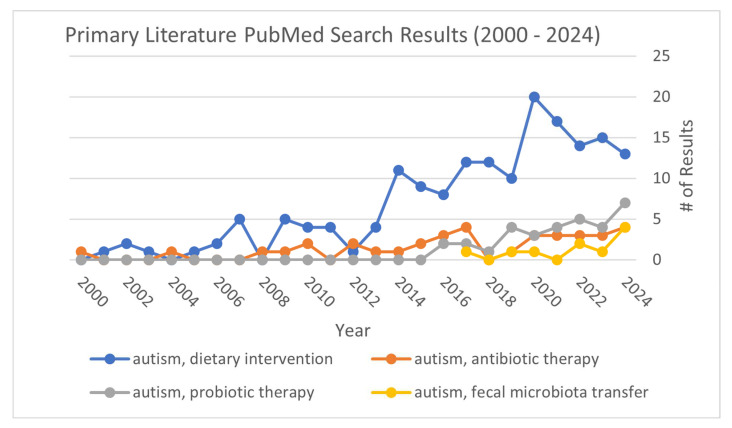
The temporal trend in manuscripts addressing different microbial interventions in autism research. Literature search of primary research papers is plotted in Figure 1, excluding reviews and commentaries, in the PubMed database for the following search terms: “autism and probiotic therapy” (grey), “autism and fecal microbiota transplantation” (yellow), autism and “antibiotic therapy” (orange), and “autism and dietary intervention” (blue). “Autism and dietary intervention” had the most search results out of all the terms searched.

Given the importance of the gut microbiota in host physiology and health, and its influence on the central nervous system via the microbiota–gut–brain axis, it has become a popular therapeutic target for neuropsychiatric disorders (Figure 1). Therapeutic approaches targeting the gut microbiota in ASD are of great interest, as they are relatively easy to administer and non-invasive. Dietary supplementation of pre- and probiotics, fecal microbiota transplantation (FMT) therapy, and dietary changes as well as antibiotic treatments, have all been used as potential treatments in ASD for GI symptoms and aberrant behaviors. Below, we provide an updated overview of microbial-based interventions used to treat the behavioral aspects of ASD and highlight articles that consider the impacts of interventions on ASD-related gastrointestinal conditions (Table 1). Our focus has been on children with ASD and GI issues; however, not all studies report GI changes post treatment or have established a baseline of GI issues, with reported clinical details being limited for some studies. An outstanding question within the field is whether the microbiome is equally disrupted in individuals with ASD with or without GI issues, and whether dysbiosis can occur in the context of ASD in the absence of profound GI problems.

## 2. Antibiotics

Sandler and colleagues were among the first to document the use of antibiotics to correct ASD-related microbial dysbiosis [10]. In their seminal study, 11 ASD children were treated with vancomycin 23 times a day for 8 weeks, followed by 4 weeks of probiotic treatment of *Lactobacillus acidophilus*, *L. bulgaricus*, and *Bifidobacterium bifidum* in an open-label trial [10]. Despite short-term behavioral improvements, long term benefits were non-apparent, with some subject’s behavioral improvements returning to baseline no less than 2 weeks after treatment termination, a time period much less than most placebo effects [11]. Since then, a few case reports have documented the use of metronidazole, amoxicillin, cefazolin, and Bactrim (medication brand) and reported corresponding behavioral improvements, though again short-lived, in ASD children [12,13]. Antibiotic usage to treat other neuropsychiatric conditions are mixed, as a recent systemic review evaluating the efficacy of antibiotics for schizophrenia found little significant impact [14]. In fact, antibiotic treatment itself has been associated with adverse neuropsychiatric events [15]. There are several reasons why antibiotic therapy in ASD may not be the most appropriate approach, including antibiotic resistance; however, these early works were important in establishing the connection in treating ASD symptomology via microbial manipulation.

## 3. Dietary Interventions

Several dietary intervention strategies have been proposed to help ameliorate GI and behavioral symptoms in ASD, with the most common interventions including casein-, gluten-, and/or carbohydrate (ketogenic)-free diets [16]. Implementing dietary interventions in young children with ASD may be challenging due to textural/sensory sensitivities, food aversion, and/or food restriction. Consequently, the nutritional profile of a child is often limited, and the microbiome is shifted towards one that is associated with a diet that is impacted by such food aversions/restrictions [17]. In a recent national survey polling 818 caregivers and adults with ASD, a healthy diet was associated with an overall benefit, with gluten and casein-free (GFCF) and ketogenic diets being among the most popular dietary form of intervention, and also the ones with the largest perceived benefit [18]. Both GFCF and ketogenic diets were reported to improve cognition and social interaction and understanding; however, only the ketogenetic diet improved constipation and seizures [18]. Similar findings were observed in an open-label clinical trial, where 15 ASD children were given a ketogenic gluten-free diet with medium-chain triglycerides (MCT) [19]. Autism Diagnostic Observation Schedule-2 (ADOS-2) cumulative scores were reduced 3 months after the modified ketogenic diet introduction, as well as observing a reduced ADOS-2 social affect. Several measures on the CARS-2 assessment were also improved, including imitation, body use, and fear and nervousness. Lastly, a modified ketogenic diet reduced BMI and the percentage of peripheral eosinophils [19]. Increased eosinophil infiltration into the duodenum has previously been shown in ASD, with a recent meta-analysis reporting a relationship between eosinophilic GI disorders and ASD [20,21]. Whether similar decreases in intestinal eosinophils occur after ketogenic diet in ASD has so far not been tested.

Regarding GFCF diets, a recent meta-analysis evaluating the effectiveness of GFCF diets across 8 studies found that stereotypical behaviors and cognition were significantly improved, but not social and communication issues [22]. Other studies have found no differences or profound improvements after GFCF diets on ASD behaviors [23,24]. It is hypothesized that the benefits from exclusion diets of this nature are related to excess opioids, where gluten- and casein-derived peptides (gliadamorphine and casomorphine) that are functionally similar to opioids act on the central nervous system to exacerbate ASD symptoms [25]. Increased IgA and IgG antibodies targeting proteins found in whey and cow’s milk have been observed in ASD; therefore, it is also possible that the elimination of gluten- and casein-derived antigens improves behavior by reducing the severity of antibody-mediated food allergies [26].

### 3.1. Prebiotics

Prebiotics are non-digestible compounds that benefit the host by supporting beneficial microbial growth [27]. Carbohydrates and oligosaccharides are the largest class of prebiotics, though other non-carbohydrates and dietary-related compounds can also be considered prebiotics, such as plant-derived fibers, guar gum, and resistant starches [27]. The health benefits of dietary supplementation with prebiotics have been observed to reduce severity in a variety of health conditions, including those characterized by GI issues. For instance, in individuals suffering from constipation, short-term supplementation with partially hydrolyzed guar gum (PHGG) results in increased bowel movements and beta diversity of bacterial populations [28]. Moreover, daily supplementation for two months with PHGG resulted in an increased abundance of *Bloutia* and *Acidaminoccus* spp. and improved irritability [29]. PHGG also reduced GI symptoms and lowered serum levels of the proinflammatory cytokine IL-1β. Prebiotics also impact health in neuropsychiatric disorders. For instance, fructo-oligosaccharides (FOS) and galacto-oligosaccharides (GOS) have anti-depressant effects, likely though reducing cortisol levels and inflammation [30].

In ASD, the supplementation of prebiotics has generally resulted in improved behaviors, reduced quantities of potentially pathogenic microbes, and lower inflammatory markers. In a recent randomized control trial (RCT) led by Girimaldi and colleagues, supplementation with the beta-galactooligosaccharide mixture B-GOS, in combination with a gluten- or casein-free diet, resulted in improved sociability scores on the Autism Treatment Evaluation Checklist (ATEC) and a trend towards reduced GI symptoms [31]. Improvements in behavior and inflammation may be due to the influence of B-GOS on bacterial populations. In a continuous culture system cultured with the fecal microbiota from ASD children, supplementation with B-GOS resulted in increased *Bifidobacteria* spp. [32]. Promoting the growth of beneficial microbes can positively impact behavior, as the administration of *Bifidobacterium longum* is associated with increased social resiliency [33]. In another study, the daily administration of AFO-202, a glycan produced by *Aureobasidium pullulans*, alongside L-carnosine and behavioral therapy, resulted in significant behavioral improvement and microbial shifts/re-adjustment [34]. Lower abundances of *Enterobacter* and *Desulfovibrio* species and increases in *Faecalibacterium prancer*, *B. longum*, and the CAG:124 *Bacillota* member were also found. In a separate study testing the efficacy of bovine colostrum product (BCP) and *B*. *Infantis* treatment, BCP alone resulted in improved abhorrent behaviors, reduced GI symptoms, and reduced frequencies of TNFα^+^CD8^+^ T cells [35].

### 3.2. Probiotics

Probiotics are live microorganisms that, when ingested in sufficient amounts, can confer health benefits [27]. Standard probiotic treatment includes the administration of specific bacterial strains. The most common types are *Lactobacilli* and *Bifidobacteria*. Generally speaking, probiotics occupy microbial niches or utilize nutrients that otherwise would have supported pathogenic bacterial populations [36]. They also directly promote GI homeostasis by producing factors that promote anti-inflammatory immune cell phenotypes indirectly by promoting GI barrier integrity [37].

Microbial dysbiosis and its resulting microbial-derived metabolites are implicated in ASD etiology and severity [38,39]. In ASD, probiotic supplementation helps balance out microbial dysbiosis and results in improved behavioral outcomes. In a study by Tamavo et al., the administration of *Lactobacillus*, *Bifidobacteria*, and *Streptococcus* reduced overall *Bacillota* and *Desulfovibrio* [40]. The administration of single-strain probiotics has also generated beneficial results, with improvement in ASD-related behaviors. For example, daily supplementation with *Lactobacillus plantarum* PS128 in 131 ASD children from Italy generally resulted in increased attention span, communication skills, and autonomy, but with no significant improvements in GI symptoms [41]. In a double-blind placebo controlled study of 36 Taiwanese ASD children, supplementation with *L. plantarum* for 4 weeks resulted in minimal behavioral improvements, with the largest improvements being related to anxiety and rule-breaking behaviors, with many of the improvements also related to participant age [42]. Minimal behavioral improvements were also observed in another RCT using *Lactobacillus reuteri* in Italian ASD children [43]. Differences in study outcomes may be due to geographical location, since probiotic usage may be more effective for microbiomes from one area/region/country than in those from other countries [44]. Alternatively, minimal behavioral improvements after probiotic supplementation may indicate that the microbiome is not a primary driver of ASD behavior at young stages of life.

In model systems, oral supplementation with *Bacteroides fragilis* effectively reduced repetitive behaviors, anxiety, and sensorimotor gating issues in a mouse model of altered neurodevelopment induced by maternal immune activation during pregnancy [45]. In Shank3 KO mice, which have several ASD-relevant behaviors due to synaptic dysfunction and regulation, as well as gut dysbiosis and increased intestinal inflammation, treatment with *L. reuteri* improved sociability and repetitive behaviors in male mice [46,47]. Improved behaviors were not mediated by changing the specific composition of the gut microbiota but rather through stimulation of the vagal nerve and subsequent signaling of oxytocinergic social reward systems [47]. Targeting of the vagal nerve/oxytocin pathway may explain why the dual administration of intranasal oxytocin and *L. plantarum* S128 resulted in improved behavioral and social domains, and lower levels of the inflammatory markers SB100 and IL-1β, in recent placebo-controlled RCT [48]. Changes in the levels of proinflammatory cytokines may have direct impacts on brain activity, as some cytokines can modulate neuronal function [49]. Behavioral improvements after probiotic usage may also be due to changes in brain hyperactivity, which was previously found to be improved in ASD children after six months of probiotic supplementation with VISBIOME— proprietary blend of *Lactobacilli*, *Bifidobacterium*, *Streptococcus thermophilus*, and starch [50].

Probiotics and/or the metabolites they produce can have effects on local gut physiology, including mucosal immune responses. Probiotic intake commonly results in increases in beneficial metabolites, such as short chain fatty acids (SCFA) [51]. Butyrate, a SCFA commonly produced by commercial probiotic strains, promotes the development of colonic regulatory T cells (cT_regs_) that help maintain homeostasis and the balance between inflammatory and regulatory mechanisms, thus making butyrate a potent anti-inflammatory metabolite at mucosal surfaces [52]. Inflammation often exacerbates pre-existing GI issues, which are prevalent in ASD [53]. After probiotic supplementation with 3 *Lactobacillus*, 2 *Bifidumbacteria*, and 1 *Streptococcus* strains, lower fecal TNFα levels were observed in ASD children, which was also associated with fewer GI symptoms [40]. In a study by West and colleagues, daily supplementation with *Lactobacillus*, *Bifidobacteria*, and *L. ramus* lysates resulted in improved diarrhea and constipation symptoms, as well as improvements in all behavioral domains in the ATEC [54]. More recent studies have confirmed similar findings. In a crossover trial led by Arnold and colleagues, the administration of VISBIOME, a probiotic mixture containing *Lactobacilli* and *Bifidobacteria* species, resulted in improved scores on the pediatric quality of life inventory GI module (PedsQL-GI) that persisted for eight weeks after administration [55]. However, health benefits from consuming probiotics may be limited to ASD individuals experiencing GI issues. In a six-month RCT testing the efficacy of VISBIOME in 31 ASD children, no significant behavioral improvements were noted in the group as a whole [56]. However, when GI status was considered, VISBIOME supplementation resulted not only in GI improvements but significantly more behavioral improvements than in the children without GI issues. The variability in findings in probiotic studies may be attributed to co-occurring GI conditions and small sample sizes that prevent comparison between ASD children with or without GI symptoms. ASD children with GI issues have been reported to have more impaired speech sociability and lower cognitive and sensory awareness [57].

### 3.3. Synbiotics

The combined use of prebiotics and probiotics, referred to as synbiotics, can have greater health benefits than pre- or probiotics used alone. Synbiotics aid in the survival and stability of probiotic species by providing nutritional niches or protecting the bacteria from low gastric pH [27]. In this way, the health benefits of the probiotics can be prolonged. The study of symbiotic therapies for ASD has increased and can overcome some disadvantages of other microbial-based therapies previously used for ASD [10]. It may also explain why in some studies, the administration of prebiotics or probiotics alone has fewer therapeutic benefits than combined [58].

There is evidence that suggests synbiotic therapy improves ASD symptomology. In a recent trial testing the efficacy of the combined probiotics *Bifidobacterium* and *Lactobacillus* species and prebiotic FOS in 26 ASD children, improved ATEC scores in speech–language communication and sociability were noted 60 days after beginning supplementation [59]. Reduced abundances of *Clostridium* and *Ruminococcus* as well as increased levels of fecal SCFA acetate, butyric, and propionic acid were also observed. Furthermore, using the Simulator of the Human Intestinal Microbial Ecosystem (SHIME) gut culture system, synbiotic supplementation with GOS plus *L.* *reuteri*, *B. longum* in the fecal microbiota from ASD children, reduced the abundance of *Desulfovibrio* and *Bacillota*, while increasing the abundance of beneficial microbes like *Lactobacillus*, *Bifidobacterium*, and *Blaotia* spp. [60]. Increases in SCFA, namely, acetic and butyric acid, were also noted. The SCFA findings of Duque and colleagues’ contrast those of Adams et al., who observed lower total SCFA content in ASD children taking probiotics [57,60]. Nevertheless, SCFA can have therapeutic potential when properly balanced, with an imbalance in either direction being undesirable.

While synbiotic microbial and prebiotic supplementation generally results in improved behavior, inconsistencies regarding the extent of their benefits exist. For instance, a pilot study by Sanctuary and colleagues found no therapeutic advantage of using a symbiotic treatment on behavior but did show improvement in GI symptoms [35]. However, their relatively small sample size (8 ASD subjects) and lack of controls limits interpretation of the efficacy of symbiotic therapy in ASD. Inconsistencies may also present because no signature panel of microbes or metabolites has been observed in microbial dysbiosis in the context of ASD. Addressing an imbalance is therefore often personalized and not easily translated to the ASD population as a whole or even to subgroups within the ASD spectrum or based on specific comorbidities. Therefore, supplementation with one type of pre-, pro-, or symbiotic does not likely address inter-individual variation in gut dysbiosis. Precision biotics may be essential for overcoming these obstacles. In a new clinical trial using the Sun Genomics product Flore, the customized administration of pre- and probiotics in 296 ASD individuals for three months improved receptive and expressive language, cognitive scores, and decreased overall GI symptom severity [61].

## 4. Fecal Microbiota Transplantation

Despite the therapeutic benefits that come with antibiotic and probiotic treatment, their efficacy to manage microbial dysbiosis in ASD is limited. Fecal microbiota transplantation (FMT) therapy, first approved for the treatment of *Clostridioides difficile* infection in 2022, has been used to successfully treat GI conditions caused by microbial dysbiosis, such as ulcerative colitis (UC) and Crohn’s Disease (CD) [62]. More recently, FMT therapy has been considered for conditions originating outside of the GI tract, including neurological conditions and neurodevelopmental disorders, including ASD. In a now seminal clinical trial from Kang and colleagues published in 2017, FMT therapy using standardized human gut microbiota (SHGM) and its impact on ASD-related behavior and GI symptoms was tested in 18 ASD children with GI symptoms [63]. Using a combination of vancomycin treatment, high dose rectal and oral SHGM administration, and maintenance SHGM doses for 10 weeks afterwords, followed by an 8 week observation period, significant improvements in ASD behavior and GI assessment scores were apparent observed by week 10, and continued for 8 weeks after treatment [63]. Behavioral and GI symptom improvements were followed by shifts in the abundance of *Bifidobacterium*, *Prevotella*, and *Desulfovibro* members. In a separate open-label clinical trial with 40 ASD participants with GI issues, FMT therapy was administered for 4 weeks without prior antibiotic treatment, followed by an 8-week observation period [64]. Gastrointestinal Symptom Rating Scale (GSRS), ABC, and CARS scores improved after the 4-week period FMT administration and remained improved until the end of the study observation period [64]. In ASD participants who responded to FMT therapy versus those who did not (defined by having less than a 50% reduction in average GSRS score following FMT treatment), *Eubacterium coprostanolegenes*, a member of the *Bacillota* phyla, was significantly reduced in the ASD responders after therapy [64]. Since these initial studies, several recent studies have also suggested positive outcomes after FMT treatment. In a study by Li and colleagues, administration of lyophilized donor stool of FMT orally once every 4 weeks (with a 12-week study end point) resulted in improved behavioral (ABC and CARS) and GI (GSRS) symptoms [65]. Similarly to Kangs 2017 study, the abundance of bacterial species changed following FMT treatment, while diversity did not [65]. An increased abundance of the genera *Eubacterium*, *Anaerostipes*, *Fusicatenibacteria*, *Collinsella*, and *Dorea* was noted, whereas *Blautia*, *Prevotella*, and *Sellimonas* were decreased 12 weeks after treatment [65]. Recent case reports of FMT treatments in ASD individuals also report significant behavioral improvements [66,67]. Changes in the abundance of microbial community members can impact the metabolomic profile of both the gut and peripheral blood, which may also contribute to behavioral improvements [68]. Before FMT therapy, plasma metabolites involved in energy metabolism and anti-oxidation were distinctly lower in ASD children, most notably including nicotinamide riboside, indolepropionate, methylsuccinate, inosine monophosphate, and sarcosine. After FMT therapy, these metabolites were similar in concentration to controls [69]. A similar pattern of changes occurs in fecal samples, where the metabolites of ASD children become more like control children, although the effect is less pronounced [70]. Serum levels of neurotransmitters that are also dysregulated in ASD, such as serotonin (5-HT), gamma-aminobutyric acid (GABA), and dopamine (DA), were also improved after FMT [64].

**Table 1:** Major clinical trials evaluating the efficacy of different microbial manipulation methods in ASD.

Author	Method	Therapy	Study Length	N	Behavioral Outcomes	GI Changes	Microbiome Changes	Other Changes	Ref.
Sandler et al., 2001	Antibiotic	Vancomycin	8 weeks, 3× daily	ASD = 11 (3– 7years)	↑ Communication and behavior	N/A	↓ Anaerobic cocci	Behavioral improvements diminished within 2 weeks of treatment	[10]
Inoue et al., 2019	Prebiotic	PHGG	2–15 months	ASD = 13 (4–9 years)	↓ Irritability	↓ Constipation	↓ α-diversity ↓ *Streptococcus*, *Odoribacter*, *Eubacterium* ↑ *Blautia*, *Acidaminococcus*	↓ IL-1β, IL-6	[29]
Grimaldi et al., 2018	Prebiotic	B-GOS and/or dietary intervention	6 weeks, daily	ASD = 13 (5– 10 years)	A trend towards improved sleep patterns ↑ Social behavior ↓ Antisocial behavior	Children on exclusion diets had improved abdominal pain and bowel movements	↑ *Bifidobacterium* spp., *Ruminococcus* spp., members of *Lachnospiraceae*, *Eubacterium dolichum*, *TM7-3* family and *Mycobacteriaceae*.	Positive associations with B-GOS intake and ethanol, DMG and SCFA metabolites Negative associations between B-GOS intake and amino acids and lactate	[31]
Tomova et al., 2014	Probiotic	Mixture of *Bifidobacteria*, *Lactobacillus*, and *Streptococcus*	4 months, 3× daily	ASD = 10 (2–9 years) ASD siblings = 9 (5–17 years) TD = 10 (2–11 years)	N/A	N/A	↓ *Bacteroidetes*, *Bacillota*, *Bifidobacterium* spp. and *Desulfovibro* spp.	↓ Fecal TNFα Positive association between GI symptoms and behavior Association between *Desulfovibro* and restrictive/repetitive behaviors	[40]
Mensi et al., 2021	Probiotic	*Lactobacillus plantarum* PS 128	6 months	ASD = 131 (mean age = 7)	Improved clinical global impression scores	N/A	N/A	There was an association between younger age and probiotic-mediated behavioral improvements	[41]
Liu et al., 2019	Probiotic	*Lactobacillus plantarum* PS 128	4 Weeks	ASD = 71; 39 Placebo: 36 PS128 (7–15 years)	↓ Body and object use, SRS-total, anxiety, rule-breaking behaviors, SNAP-IV total scores, hyperactivity, and impulsivity (exploratory analysis only)	N/A	N/A	Behaviors improved more in 7–12-year-old children	[42]
Billeci et al., 2022	Probiotic	VISBIOME—a mixture of *Lactobacilli*, *Bifidobacterium*, *Streptococcus thermophilus*, and starch	6 months	ASD receiving probiotic = 26 ASD receiving placebo = 20	↓ Frontopolar power ↑ Beta and gamma waves in the frontopolar coherence (concentration and working memory)	N/A	N/A	Negative correlation between frontopolar coherence and peripheral TNFα Decreased power related to decreased RBS-R total scores Increase in coherence related to increased VABS-II “Writing skills	[48]
West et al., 2013	Probiotic	Delpro Supplement—a mixture of *Lactocillus*, *Lactobacillus*, and *Bifidobacteria* strains and Del-Immune V powder derived from *L. rhamnousus*	21 days, 3× daily	ASD = 33 (3–16 years)	↓ ATEC scores (speech/language, sociability, sensory/cognitive awareness, and physical behavior)	↓ Constipation ↓ Diarrhea	N/A	Several caregivers reported that it would take longer than 21 days to see improvements Caregivers also reported the “immunity booster” in the Delpro supplement seemed to make a difference compared to their last probiotic experiences	[52]
Arnold et al., 2018	Probiotic	VISBIOME—a mixture of *Lactobacilli*, *Bifidobacterium*, *Streptococcus thermophilus* and starch	19 weeks (8 weeks daily treatment followed by 3 weeks washout then 8 weeks cross-over daily treatment)	ASD = 10 (3–12 years)	Trend of improved ABC irritability, ABC hyperactivity, PSI total stress and CSHQ assessments	Trend of improved PedsQL GI total score	No changes in α-diversity	↑ % of *Lactobacillus* associated with improved Peds QL Scores	[53]
Kang et al., 2017	FMT	2-week vancomycin treatment followed by fecal microbiota transfer (1 initial high rectal or oral dose, followed by daily, oral, maintenance doses)	18 weeks (10-week treatment, 8-week observation)	ASD = 18 (7–16 years) TD = 20 (age/sex matched, no treatment)	↑ Increased total scores on the CARS, SRS, ABC, and VABS-II assessment	↓ Abdominal pain, indigestion, diarrhea, and constipation ↓ GSRS scores	↑ Bacterial diversity ↑ *Bifidobacterium*, *Prevotella*, and *Desulfovibro*	No difference between oral or rectal initial doses Bacteriophage richness and evenness were largely unchanged following treatment	[61]
Li et al., 2021	FMT	Weekly FMT, rectal or oral, therapy for 4 weeks No vancomycin or additional medication given prior to treatment	12 weeks (4 weekly treatment/8-week observation)	ASD = 40 (3–17 years) TD = 16 (age/sex matched, no treatment)	↓ CARS, SAS, and SRS total scores	↓ Hard, soft, and abnormal stools	No changes in α-diversity Reduced uniFrac distances between ASD and donor Lower *Eubacterium coprostanoligenes* in FMT responders	↓ 5-HT, GABA, DA	[63]

5-HT: serotonin; ABC: aberrant behavior checklist; ASD: Autism Spectrum Disorder; ATEC: Autism Treatment Evaluation Checklist; B-GOS: biologically active galacto-oligosaccharides; CARS: Childhood Autism Rating Scale; CSHQ: Child Sleep Habits Questionnaire; DA: dopamine; DMG: dimethylglycine; FMT: fecal microbiota transplantation; GABA: gamma-aminobutyric acid; GI: gastrointestinal; GSRS: gastrointestinal symptom rating scale; IL: interleukin; N/A: not applicable; PedsQL: Pediatric Quality of Life Inventory; PSI: Parenting Stress Index; RBS-R: Repetitive Behavior Scale-Revised; SAS: Social Affective Scale; SRS: Social Responsiveness Scale; SCFA: Short-Chain Fatty Acids; SNAP-IV: Swanson, Nolan, and Pelham-IV-Taiwan version; TD: typically developing; TNF: Tumor Necrosis Factor; VABS-II: Vineland Adaptive Behavior Scales, Second Edition.

Although the results from FMT are encouraging, an 8–10-week observation period is associated with moderate placebo effects in functional GI disorder studies [71]. Unique to FMT therapy in ASD, and no other mode of microbiome therapies, is that these positive effects of FMT persisted for a significant period outside of the study period. In a follow-up study with the participants from the Kang et al., 2017 study, behavioral (as determined by the ABC, CARS, and Vineland Adaptive Behavior Scale [VABS] assessments) and GI symptoms (determined by the GSRS) were determined. It was shown that 2 years post treatment, persistent behavioral improvement and GI symptom remission could be detected [72]. Intriguingly, some improvements were enhanced even further than at the end of the original study, and CARS scores were 47% lower after 2 years follow-up compared to 23% lower after the initial study ended [72]. Such long-term improvements may be due to increased abundances of *Bifidobacterium*, *Prevotella*, and *Desulfovibro* found during the follow-up period [72]. Furthermore, a longitudinal study evaluating the long-term outcomes in 328 Chinese ASD children found that improvements in behavioral and GI symptoms could be seen 2–3 years post-FMT treatment, but were largely lost 5 years post-FMT [73]. These encouraging results warrant further study, and investigations also need to be conducted to determine the long-term effects of other microbial manipulation strategies that so far have been lacking in the literature.

### Early-Life Microbial Interventions

Several avenues of research remain to be investigated in terms of the therapeutic potential of microbial manipulation in ASD. More recently, microbial supplementation during critical periods of brain development holds promise. In the maternal immune activation (MIA) model of altered neurodevelopment, supplementation with the synbiotic combination of *Bifidobacteria*, *Lactobacillus*, *FOS*, and *maltodextrin* during gestation protects the MIA offspring from ASD-like behaviors, likely by reducing levels of IL-17A and IL-6 in the brain and promoting the differentiation of inhibitory neuronal cells [74]. In cross-fostering experiments where MIA offspring are exposed to the microbiota of healthy dams during the early post-natal period, behaviors are also rescued, with reduced inflammation also seen [38]. In the idiopathic BTBR mouse model of ASD, the administration of sodium butyrate in utero and in adulthood reduces ASD-like repetitive behaviors and increases sociability [75,76]. In a human trial investigating probiotic supplementation during pregnancy, probiotics were associated with a reduced risk of neuropsychiatric disorders like ADHD and ASD [77]. However, no difference was found in a more extensive study testing probiotic supplementation from 35 weeks gestation until two years of age [78]. Moreover. Microbial-induced imbalances in SCFA may also have adverse effects on neurodevelopment. For instance, exposure to moderate propionic acid concentrations shifts neuronal development towards excitatory phenotypes and increases inflammatory gene expression in human neural stem cells [79].

The establishment of the early-life microbiome is directly tied with the development of immune tolerance, and because immune dysregulation is a common finding in ASD, microbial manipulation that targets immune education may be a unique therapeutic opportunity [3,80]. Early-life exposure to gut dysbiosis, such as maternal antibiotic administration during labor, delivery method (e.g., cesarean section), and diet (e.g., formula), are all associated with ASD outcomes and negatively impact the development of immune tolerance in offspring [81,82,83,84]. The importance of the gut microbiota in tolerance development is observed in germ-free (GF) mice, which lack gut microbial communities [85]. GF mice have a reduced thymus size and cellularity, and a reduced expression of the autoimmune regulator (AIRE) transcription factor (reviewed in [86]). AIRE expression in the thymus is essential for the development of regulatory T cells (T_regs_), a critical component involved in immune tolerance, and decreased frequencies and function of these cells are also implicated in ASD pathology. Failure to express AIRE results in autoimmunity and inflammation [87]. In pregnant and lactating dams fed a high-fiber diet, offspring had elevated serum levels of butyrate, resulting in increased AIRE expression and T_regs_ frequency [84]. This may be related to butyrate’s ability to epigenetically modify transcriptional sites necessary for T_regs_ development [52,88]. In human cohorts of expectant mothers, elevated maternal serum acetate during gestation positively correlates with thymus size and offspring T_regs_ frequency several years after birth [89]. Mechanistically, this may also be due to histone modifications at essential T_regs_ promoter sites. In mice, maternal acetate was also found to suppress allergic airway disease in offspring by enhancing T_regs_ development via acetylation at T_regs_ promoter sites [90]. Similar results have been seen with early life probiotic administration/exposure. The administration of *Bifidobacterium breve* to preterm infants resulted in increased serum levels of TGF-β1, a regulatory cytokine largely associated with T_reg_ activity [91,92].

## 5. Future Directions

There are several factors that should be considered for future therapeutic trials. Research studies of this nature should focus on recruiting study populations that accurately reflect the ASD population, particularly regarding the diversity of GI symptoms and level of behavioral needs. Moreover, the characterization of study population, including behavioral, GI status, immune status/activation, biomarkers for intestinal integrity, and additional co-morbidities, will assist in the interpretation of trial results. The duration of potential microbiome manipulation approaches and the desired follow-up periods warrants further exploration. Consideration to trial design and whether a combination of therapies (e.g., probiotic use and fecal microbiota transplantation) is more effective than any single approach, as some citied studies suggest a synergistic effect when two or more microbial manipulations were used (i.e., antibiotic therapy and FMT), compared to a minimal effect when one was used (i.e., one probiotic strain). Moreover, it is crucial to investigate the timing of interventions, particularly the differences between early childhood and adolescence, as these are sensitive time periods in the development of the gut microbiota. So far, most trials have been limited to small populations, without typically developing or sibling controls. Lastly, while there are some benefits that come with traditional microbial intervention strategies and treatments, future studies should also consider the development of non-traditional approaches to microbial manipulation, such as personalized microbiome therapies, novel drugs, or novel dietary strategies. Still unknown are the therapeutic effects in children with ASD who present with GI issues compared to those without GI issues. Answering which populations are best served by microbiome manipulation will be crucial going forward. In summary, to build confidence in trial outcomes when no or minimal behavioral improvement is observed, study populations need to be well defined and the biology of the microbiota—as it pertains to both the host and microbiome itself as an organism—need to be carefully considered.

## 6. Conclusions and Limitations

**Figure 2:**
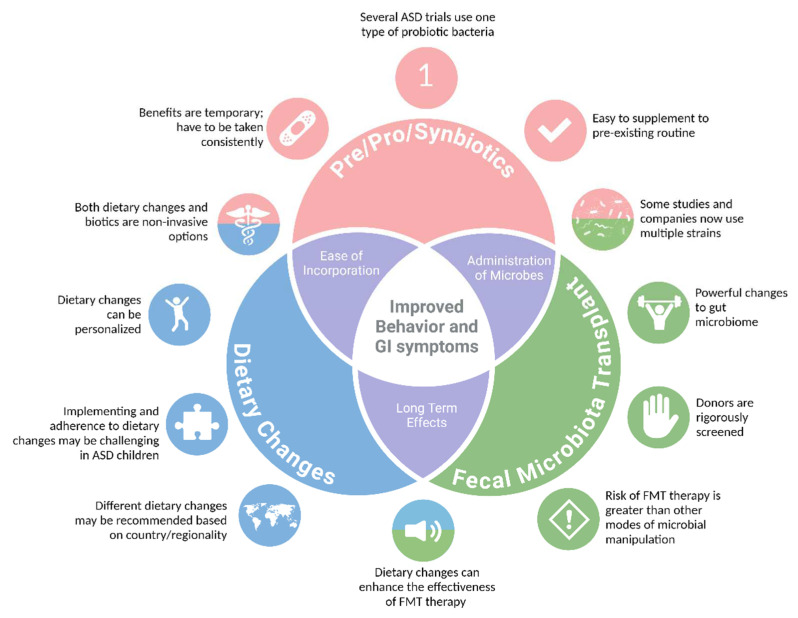
Similarities and differences in microbial-based therapies used in ASD. A variety of microbial-based therapies have been explored for autism spectrum disorder (ASD), with the most common methods including probiotics (along with prebiotics and synbiotics), dietary changes, and, more recently, fecal microbiota transplantation (FMT). Each method has unique advantages—such as the ease of use of probiotics and the potent effects of FMT. These therapies share characteristics, and in some cases, they may demonstrate enhanced therapeutic effects when administered in combination rather than individually. Moreover, their combined use may prove to hold greater and longer-lasting behavioral and gastrointestinal improvements in individuals with ASD.

Manipulating the gut microbiota presents a promising avenue for modulating certain ASD behaviors and associated comorbidities (Figure 2). There are multiple ways that the gut microbiota can be targeted to treat ASD-related behaviors and co-morbidities; however, the efficacy varies. Given the fact that the composition of the gut microbiota normally changes daily, many of the therapeutic modalities discussed in this review would require daily supplementation. This may be difficult for caregivers and individuals with ASD, given the restricted food interests or aversions that are present in ASD. Other modalities, such as FMT, appear to hold promise. However, given their invasive nature—both during FMT preparation and administration—application may be difficult for the individuals and/or their families. An additional consideration for treatment approaches includes subject age. Most studies cited in this review have recruited children and adolescents with ASD—often combining young children and late teens into one study group. There are multiple periods in the first two decades of life where the gut microbiome is dynamic and changes in accordance with development, especially during the first few years of life. It is possible that some of the limited behavioral improvements from microbial interventions may be from the result of using a wide age gap. Age-dependent limitations may also explain the limited efficacy of popular microbial interventions, including probiotics. Another limitation for interpreting the effectiveness of microbial interventions in ASD-related conditions is the absence of well-characterized GI issues in study populations. Not all microbial intervention studies included information pertaining to GI improvements. Some of these limitations can be addressed through the development of prospective studies that follow at-risk infants throughout development, such as the proposed Genome, Environment, Microbiome and Metabolome in Autism (GEMMA) study [93].
